# Chronic Obstructive Pulmonary Disease in Never-Smokers—A Distinct Entity Within the COPD Spectrum

**DOI:** 10.3390/life16010043

**Published:** 2025-12-26

**Authors:** Andreea-Nicoleta Mălăescu, Florin-Dumitru Mihălțan, Ancuța-Alina Constantin

**Affiliations:** 1 Department of Cardio-Thoracic Pathology, “Carol Davila” University of Medicine and Pharmacy, 050474 Bucharest, Romania; florin.mihaltan@umfcd.ro (F.-D.M.); ancuta-alina.constantin@umfcd.ro (A.-A.C.); 2 Institute of Pneumology “Marius Nasta”, 050159 Bucharest, Romania

**Keywords:** COPD, chronic obstructive pulmonary disease, never-smokers, pollution, tuberculosis, A1ATD

## Abstract

Although smoking is the main risk factor for chronic obstructive pulmonary disease (COPD), about one-third of patients have never smoked. This phenomenon supports the idea of a distinct phenotype of the disease in never-smokers, influenced by genetic, infectious, socioeconomic, environmental, and occupational factors. The paper is based on a narrative review of recent literature on the etiology, clinical features, evolution, and therapeutic strategies of COPD in never-smokers, mainly through the analysis of published studies over the last 3 years. COPD in never-smokers occurs predominantly in women, the elderly, and individuals from rural areas or with poor socioeconomic status. Key risk factors include exposure to occupational or environmental pollutants, air pollution, previous respiratory infections, particularly due to pulmonary tuberculosis, and genetic predisposition, mainly through alpha-1 antitrypsin deficiency (A1ATD). Clinically, COPD in never-smokers is characterized by chronic cough and dyspnea, with less severe pulmonary functional impairment, slow progression, and lower prevalence of emphysema compared to smokers. Imaging often highlights bronchiectasis or post-infectious sequelae, and biological markers indicate a significant eosinophilic component. Thus, COPD in never-smokers is a distinct clinical entity with multifactorial pathogenesis and distinct clinical-functional characteristics. Prompt recognition of this form of disease is essential for prevention and adaptation of therapeutic strategies. A personalized multidisciplinary approach can improve disease prognosis and the quality of life for these patients.

## 1. Introduction

Chronic obstructive pulmonary disease (COPD) is one of the most common causes of morbidity and mortality worldwide [1]. Alongside lung cancer, it is known as an important consequence of tobacco smoking and is often described to patients as “the smoking disease”. Although smoking is the main risk factor, about 30% of patients diagnosed with COPD have never smoked [2]. Thus, other factors that significantly influence COPD onset, including, among others, occupational or household exposure to air pollutants, a history of previous respiratory infections, or asthma, have been studied [3]. However, the existing literature on the epidemiology, risk factors, and clinical features of COPD in never-smokers remains limited.

According to the GOLD Guide (Global Initiative for Chronic Obstructive Lung Disease), COPD is a condition characterized by persistent respiratory symptoms, which result from two major pathological processes: chronic bronchitis and pulmonary emphysema. The underlying airway obstruction is confirmed spirometrically by a reduced FEV1/FVC ratio (<0.7 post-bronchodilator; FEV1 = Forced Expiratory Volume in one Second, FVC = Forced Vital Capacity), reflecting a not fully reversible obstructive ventilatory defect [1].

Recent epidemiological data indicate that the prevalence of COPD in never-smokers remains significant, ranging from 4% to 10%, which contributes substantially to the burden of this disease [4].

The clinical characteristics of COPD differ from those seen in smokers. Never-smoking patients with COPD are more frequently female, tend to be older, and have a higher body mass index. They frequently have comorbidities such as hypertension, gastroesophageal reflux disease, or osteoporosis [5]. Among never-smoking patients with COPD, there is a higher prevalence of asthma, bronchiectasis, and personal history of pulmonary tuberculosis and a lower prevalence of emphysema compared to smokers [6].

Exposure to environmental tobacco smoke (passive smoking) is an important risk factor, with a dose-effect relationship between the duration of exposure and the risk of COPD [7]. Environmental and socio-economic exposures such as air pollution, occupational risks, and low educational attainment have been identified as key factors in the development of the disease [8]. Childhood respiratory infections, asthma, and allergies are associated with a risk of developing COPD later in life, especially among women [9].

Never-smokers with COPD often have a milder course of the disease, with better lung function, fewer exacerbations, and lower mortality compared to smokers diagnosed with COPD [10]. However, the risk of exacerbations and progression of the disease, although lower, remains relevant in these patients [11].

The recognition of COPD in never-smokers highlights the need for a complex risk assessment and well-adapted management strategies addressing the etiological and clinical specificities of this population [12]. As the global burden of COPD continues to increase, understanding the mechanisms that lead to this disease in never-smokers is essential for prevention, early diagnosis, and effective intervention.

## 2. Materials and Methods

This paper is a narrative review of recent scientific literature that explores the peculiarities of chronic obstructive pulmonary disease in never-smokers. The aim was to identify and analyze current data on risk factors, clinical features, diagnostic investigations, disease progression, and therapeutic strategies associated with this distinct phenotype.

To select the sources included in this article, a literature search was conducted in major databases, such as PubMed, Scopus, and Google Scholar, in July–October 2025. The search strategy was based on keyword associations, such as “chronic obstructive pulmonary disease”, “COPD in never smokers”, “never-smoking COPD”, “biomass exposure”, “tuberculosis-associated COPD”, “air pollution”, and “alpha-1 antitrypsin deficiency”. We included articles published in English, predominantly between 2022 and 2025, consisting of original studies, meta-analyses, cohort studies, and systematic reviews.

Inclusion criteria focused on studies that:

• Included patients diagnosed with COPD according to GOLD criteria (FEV1/FVC < 0.7 post-bronchodilator);
• Included never-smokers, with no history of active smoking;
• Reported associations between never-tobacco exposures and the development of COPD;
• Compared clinical or paraclinical characteristics between never-smokers with COPD and smokers with COPD;
• Were published predominantly between January 2022 and October 2025;
• Were published in English to ensure accurate interpretation of the data.

The following were excluded:

• Studies that included only active or former smokers, with no never-smoker comparison groups;
• Articles lacking a clear description of methodological details;
• Studies presenting incomplete data;
• Papers published before 2022, except for reference articles used for theoretical background;
• Letters to the editor, editorials, and case reports.

The data extracted from the selected studies were synthesized according to their etiological and clinical implications: infectious, genetic, environmental, and occupational factors; socio-demographic characteristics; clinical manifestations; functional, imaging, and laboratory findings; comorbidities; treatment approaches; and prognosis. The comparative analysis was based on two groups: smokers with COPD and never-smokers with COPD, using the indicators reported in each study: FEV1, FVC, mMRC, CAT, inflammatory biomarkers, comorbidities, and others.

According to the present review, never-smoking COPD applies to individuals who have never smoked, defined as not being active smokers of tobacco. For the majority of the published studies, never-smokers were defined as those with a lifetime exposure to smoking of less than 100 cigarettes or less than 1 pack-year. Ex-smokers with little exposure were included only when specified as never-smokers by the original authors. Passive smoking exposure was also considered to be a relevant risk factor and is discussed separately as an etiological contributor, rather than an exclusion criterion.

The results were presented descriptively and in tabular form, focusing on the clinical, functional, and prognostic differences identified in the two groups.

## 3. Discussions

The development of COPD in never-smokers is increasingly recognized as a multifactorial process in which multiple environmental, socioeconomic, occupational, and biological factors contribute to the onset of the disease.

Lee et al. looked at potential phenotypes in COPD by analyzing two patient databases in Korea and identified that 32.6% of the overall COPD population did not have an identified risk factor [3]. The KOCOSS study (The Korea COPD subgroup study) followed, among other things, the clinical history of COPD among never-smokers. Bronchial asthma was identified in almost half of the respective patients, pulmonary tuberculosis in approximately 37%, childhood infections in around 30%, and bronchiectasis in 15% [5].

Risk factors for COPD in never-smokers can be systematically classified into several categories according to recent studies. These categories include:

1. Personal and medical history [13];
2. Socioeconomic and demographic factors [6];
3. Environmental and occupational exposures [14].

### 3.1. Personal and Medical History

Infectious factors play an important role in the development of typical COPD pathology [15].

Patients with a history of pulmonary tuberculosis represent a significant proportion of never-smokers who develop COPD; in this context, female patients appear to have a higher relative risk compared with males [3]. The exact mechanism is not clear, but several hypotheses exist [16]. One dominant theory is that structural damage remains following TB, including cavitation, fibrosis, bronchiectasis, and airway stenosis, which leads to fixed airflow obstruction and small-airway dysfunction that is clinically indistinguishable from classical COPD even years following microbiological cure [17]. A secondary perspective concerns persistent and dysregulated inflammation: converging innate and adaptive immunity in TB and COPD, including neutrophil- and macrophage-mediated responses and persistent cytokine efflux, might persist after infection resolution, facilitating airway remodeling, emphysema, and a specific TB-COPD phenotype [18]. The importance of protease function and oxidative stress are closely related to each other in that over-induction of neutrophil degranulation, matrix metalloproteinases, and reactive oxygen species enhances persistent parenchymal destruction and fibrotic scarring, leading to the accelerated irreversible airflow limitation [19]. Sex-specific mechanisms have also been proposed to explain the higher relative risk levels seen in women. Estrogen-modulated inflammatory and growth pathways may enhance chronic inflammation and tissue repair abnormalities in the post-TB lung and further increase susceptibility to COPD in never-smoking females, while sex-dependent immune responses could also account for this [20]. And since sex-related morphological abnormalities in airway structure and lung development—such as smaller airway calibre for a specific lung size and contrasting trajectories of lung growth and aging in women—might exaggerate functional implications of post-tuberculous scarring and small-airway loss, resulting in worse lung function for equivalent anatomic destruction [21].

Direct destruction of lung tissue in pulmonary tuberculosis is characterized by the formation of cavities, fibrosis, and bronchiectasis. These predominantly irreversible changes cause airflow limitation, a characteristic of COPD. Post-TB fibrotic remodeling is asymmetric and predominantly affects the upper lobes, which differentiates post-TB COPD from smoking-induced COPD. The whole process of airway remodeling and fibrous scars after tuberculosis infection leads to bronchial stenosis and obliterative bronchiolitis, both of which further reduce lung function [17].

Chronic pulmonary inflammation from pulmonary tuberculosis involves both innate and adaptive immunity. Among the cells involved, macrophages, neutrophils, and dendritic cells are activated and release proinflammatory cytokines, such as TNF-alpha and IL-8, which maintain tissue damage and remodeling, even after complete treatment of tuberculosis [22]. This inflammation is the reason for the installation of airway obstruction and damage to the post-tuberculosis lung parenchyma [18]. Moreover, after effective treatment of infection, mycobacteria can persist in macrophage cells, leading to chronic inflammation and progressive damage to the airways, increasing the risk of COPD in survivors [22].

Moreover, in tuberculosis, the activity of matrix metalloproteinases (MMPs) is stimulated. These are enzymes that degrade the extracellular matrix, thus leading to the destruction of the alveolar walls and the formation of pulmonary emphysema [23]. This imbalance between MMPs and their inhibitors is the common mechanism in both pulmonary tuberculosis and COPD [22].

In addition to pulmonary tuberculosis, there are other infectious factors that contribute to the onset of COPD. A common aspect is the formation of bronchiectasis, the consequence of severe or recurrent respiratory infections. A population study in Korea identified that the presence of bronchiectasis is associated with a six-fold higher risk of COPD (OR 6.0; 95% CI: 1.4–25.4), independent of age, sex, or body mass index (BMI) [13]. The mechanisms involved involve impaired mucociliary clearance, chronic inflammation of the airways, and recurrent infections, which contribute to irreversible airway obstruction [6,13].

The history of respiratory infections in childhood, including pneumonia and other lower respiratory infections, increases the risk of developing COPD in never-smoking adults, a risk that becomes even higher when additional environmental exposures such as biomass smoke or passive smoking are present [14,24,25,26,27]. Another hypothesis is genetic susceptibility to respiratory infections in childhood, which would mean that alterations in lung development precede respiratory infections. Frequently responsible for respiratory infections in childhood are Streptococcus pneumoniae and Haemophilus influenzae, often causing severe pneumonia. Inadequate treatment in developing countries leads to a high prevalence of COPD in these regions [28].

HIV infection (Human Immunodeficiency Virus) is another important risk factor for COPD in never-smoking patients [29,30]. This condition causes persistent retention of CD8+ T lymphocytes in the lining of the airways, leading to chronic inflammation and remodeling, processes involved in COPD. This effect is observed even in the absence of smoking [31].

Alpha-1 antitrypsin deficiency (A1ATD) is a well-established genetic risk factor in the pathogenesis of COPD in never-smokers. A1ATD is caused by mutations in the SERPINA1 gene. The most severe form of deficiency is found in individuals with the PiZZ genotype, although other variants, such as PiSZ, may contribute to the risk of COPD [32]. Although never-smokers with severe A1ATD are at significant risk of developing COPD, the course of the disease is milder than in smokers. A study in Sweden found that 9% of people with severe A1ATD developed COPD during the follow-up period, compared with 16% of smokers [33].

A1ATD produces a proinflammatory state in the lungs before the onset of COPD. Individuals with this deficiency but normal lung function have an increased number of neutrophils, high protease activity, and increased levels of proinflammatory cytokines in the lower respiratory tract. Early anti-inflammatory interventions may help prevent the COPD progression [34].

A1ATD is commonly associated with panacinar emphysema. Early identification allows for early implementation of preventive and therapeutic measures [35].

### 3.2. Sociodemographic Factors

Sociodemographic factors play an important role both in the risk of developing COPD and in the course of this disease. Recent studies show the interaction between age, gender, race, and socioeconomic status in shaping the risk of COPD in the population.

The claim that the prevalence and severity of COPD increases with age is based on a well-established truth, even among never-smokers. Most studies show that COPD in never-smokers occurs predominantly in older adults, possibly because of the need for longer exposure to never-tobacco risk factors. Another explanation for the diagnosis of COPD at older ages is late diagnosis, resulting from milder symptoms of the disease in never-smokers and a lack of association with smoking [2]. Thus, the prevalence of COPD can be considered to increase considerably after the age of 60 [14].

Differences in prevalence by sex are also considerable. Although historically men were the most prevalent category of COPD, recent data have shown an increase in it among women in some geographical areas, such as Hungary, Morocco, or South Korea [36]. In never-smoking populations, COPD is considered to be more common in women (Table A1), especially in low- and middle-income countries, where indoor pollution from cooking or biomass fuels, as well as outdoor pollution, is higher [2]. In China, over three-quarters of women with COPD are never-smokers, while only a quarter of men with COPD have ever smoked [26].

Racial and ethnic disparities participate in COPD risk. Long-term exposure to air pollutants such as nitrogen oxides is associated with a higher risk of COPD in never-smokers, with more pronounced effects being identified in African-American, Latin American, and Japanese-American populations compared to white [37]. Another study, led by Wang et al., among young adults in the United States, identified a higher likelihood of developing COPD in non-Hispanic White and Black individuals, independent of smoking status, which may highlight the impact of genetic susceptibility [38]. Among Hispanic adults, low income, poor health, or lack of health insurance are associated with a high risk of COPD, outlining the importance of the social determinants of the burden of the disease [39]. However, the specialized literature did not establish exactly which race or ethnicity is more prone to developing COPD. Furthermore, racial inequities and the use of race-based spirometry equations may be indirect risk factors in the underdiagnosis and delay in initiating appropriate treatment, especially in African Americans [40].

A low socioeconomic status, which includes low educational attainment, low family income, malnutrition, and poor living conditions, is associated with a higher risk of developing COPD [41]. Moreover, the course of the disease is influenced by both individual and community income. High incomes are associated with low hospitalization and mortality rates, while individuals from rural or suburban areas have a higher risk of hospitalization [42].

### 3.3. Exposure to Respiratory Pollutants

Long-term exposure to air pollutants, which include particles such as PM2.5 (particles ≤ 2.5 μm) and PM10 (particles ≤ 10 μm), as well as nitrogen dioxide, ozone, and carbon monoxide, is associated with an increased risk of developing or progressing COPD in never-smokers, especially among women [43]. Furthermore, a study conducted by Huang et al., which tracked PM2.5 exposure in concentrations of >38.98 μg/m^3^, demonstrated a 29–69% higher risk of developing COPD in exposed individuals compared to those not exposed [44,45]. Pathogenic effects of these particles are thought to involve oxidative stress, inflammation, and airway remodeling, mechanisms that together lead to chronic lung damage and airflow obstruction [46]. Studies on large population cohorts have shown that each increase in concentrations of PM2.5, PM10, NO_x_ and NO_2_ is correlated with a higher risk of COPD [47].

Another factor associated with COPD development is living in close proximity to heavily trafficked roads. A cross-sectional observational study conducted in a province of China has proven that air pollution caused by traffic independently increases the risk of developing COPD, even after adjusting for confounding factors such as smoking status and age. In this study, led by Zheng et al., exposure was defined by the distance of housing and outdoor activities in the proximity of heavily circulated roads. Outdoor activities performed within 200 m of congested roads were associated with a significantly higher COPD risk compared with activities undertaken more than 200 m away [48]. Nitrogen dioxide, a marker of traffic emissions, has been associated with an increased incidence of COPD, being involved in airway inflammation and dysfunction of their epithelium [49].

Air pollution in homes, particularly from burning biomass fuels for heating or cooking purposes, particularly in low- and middle-income countries, leads to a significantly higher risk of COPD in never-smokers [50]. This chronic exposure results in a distinct COPD phenotype, characterized by mainly respiratory tract damage, reduced emphysema development, and slower decline in lung function compared to smoking-related disease [51].

Occupational exposure to respiratory pollutants remains one of the most important risk factors in the pathogenesis of COPD. Certain chemicals, including pesticides, heavy metals (lead, chromium, arsenic, and cadmium), and polycyclic aromatic hydrocarbons, are associated with an increased risk of developing COPD in work environments [52].

Occupational exposure to pesticides has been identified as an important risk factor for COPD, with De Matteis et al. proving that any level of exposure is associated with the onset of COPD, with a dose-response relationship, and confirmed results including smokers and individuals without asthma [53].

Exposure to vapors, gases, dust, and smoke (VGDF = vapors, gases, dusts, and fumes) is independently associated with a higher prevalence of COPD and respiratory symptoms, including work-related symptoms, even after adjusting for confounding factors such as age and smoking status. Of the people who work and are exposed to VGDF, about 8% develop COPD [54]. Occupational exposure to biological dust, gas, or smoke and solvents was associated with both COPD and pulmonary emphysema, a theory confirmed by computed thoracic tomography and spirometry in a Spanish population [55]. Another study found that of more than 7000 participants, cough and expectoration were significantly correlated with exposure to dust. Moreover, simultaneous exposure to dust and gases, vapors, or fumes was associated with an increased risk of chronic bronchitis and reductions in the Tiffeneau index (FEV1/FVC) [56].

However, the long latency period of COPD development and multiple exposures make it difficult to isolate the risk attributable to specific substances [52].

The decrease in the prevalence of tobacco smoking in a population impacts not only the decrease in the incidence of COPD in smokers but also in other individuals. Passive smoking is a well-established risk factor in COPD, with a dose-effect relationship between the duration of exposure and the risk of disease, with individuals with over 5 years of exposure having a 4.38-fold higher risk compared to those not exposed [57].

### 3.4. Clinical Picture

COPD in never-smokers is increasingly considered a distinct clinical entity, with unique symptomatology and evolution, compared to the disease occurring in smokers. In never-smokers it manifests through symptoms that may differ in frequency, severity, and impact from those in smokers.

Dyspnea is a key symptom of COPD, even in people who have never smoked. Never-smoking COPD patients experience less intense dyspnea compared to smokers, as measured by the mMRC (modified Medical Research Council) questionnaire. Patients who have never smoked reported lower mMRC scores [10]. However, in never-smokers, dyspnea remains a significant symptom, usually the reason patients seek medical attention. A prospective observational study revealed that never-smokers reported a longer duration of dyspnea prior to diagnosis. Because clinicians often have a lower suspicion of COPD in never-smokers, dyspnea develops insidiously [58].

Never-smoking women with COPD report episodes of dyspnea that are more severe and frequent than men, and also greater limitations in energy levels, daily activities, sleep, and self-confidence [59]. Low effort tolerance associated with dyspnea occurs even in mild forms of COPD, being the main factor involved in limiting daily activities [60]. In addition, more than 40% of patients report significant emotional and psychological impact, including depression, discomfort, or anxiety during episodes of difficult breathing [61]. Never-smoking COPD patients show greater improvement in mMRC scores and increased exercise activity after pulmonary rehabilitation, suggesting a better response to non-pharmacological interventions compared with smokers [62].

Another common symptom of COPD is chronic cough, which is often the reason why the patient goes to the doctor. However, the impact of this symptom in COPD is similar in smokers and never-smokers. A Korean study that included several health units found no significant differences in the intensity of cough in smokers and never-smokers, suggesting that the burden of cough is similar in COPD regardless of smoking status [5].

Coughing has a significant impact on lifestyle, interfering with sleep and social interactions. It is also an indicator of a higher risk of exacerbations and mortality, independent of smoking status or airflow limitation [63]. Of those with chronic cough, around 18% report that it affects their daily lives, and about 55% consider it a problem, especially at night or in conversations [64].

Chronic cough is also associated with a greater decline in lung function as measured by FEV1, suggesting that cough itself may contribute to the progression of COPD [65].

Expectoration is commonly associated with cough, being a marker of inflammation of the airways. When common, it is associated with a more significant history of exposure to pollutants, a greater limitation of airflow through the airways, and also with a significant risk of exacerbations and hospitalizations, regardless of smoking status [66]. The NOVELTY study identified that patients with frequent productive cough had an approximately 1.7-fold increased risk of exacerbation during 12 months of follow-up [66]. During exacerbations, a predictor of worsening of symptoms and the need for hospitalization is the increase in the amount of expectorated sputum [67].

Air pollution or temperature extremes can worsen cough severity. Low ambient temperatures are associated with increased cough and expectoration, while high temperatures can increase dyspnea [68].

Wheezing is a widespread symptom in never-smoking patients with COPD, with over half experiencing it, a rate similar to that in patients with ACO (Asthma-COPD Overlap Syndrome) [69].

The presence of wheezing in never-smoking patients with COPD is associated with more important symptoms, decreased lung function, and a higher risk of exacerbations compared to those who do not have wheezing. In a Korean cohort, COPD patients who had wheezing had greater airflow limitation and experienced more exacerbations than those without wheezing. Thus, this wheezing was shown to be a predictor of the risk of exacerbations, independent of other factors such as smoking or airflow limitation through the airways [69].

Moreover, wheezing is a basic symptom for identifying undiagnosed COPD cases, especially in never-smokers. A study in Norway demonstrated the importance of including wheezing in screening questionnaires, along with smoking history, in detecting yet undiagnosed COPD [70].

However, COPD is a disease that has effects not only on the respiratory system but also on the whole organism. Fatigue and muscle weakness are commonly reported symptoms in COPD patients regardless of smoking status. They contribute to the burden of the disease, affecting daily activities and quality of life [71].

Depression and anxiety are common in COPD patients, their severity being associated with overall symptom burden and the risk of exacerbations. In addition, these symptoms can worsen the perception of dyspnea and fatigue. They can be associated with sleep disorders, including insomnia, which clearly has a direct negative impact on quality of life [72].

### 3.5. Paraclinical Investigations

#### 3.5.1. Measuring Pulmonary Function

Important differences between smokers and never-smokers have been identified in pulmonary function tests. Never-smoking patients diagnosed with COPD usually have less severe airflow limitation and thus have a higher FEV1 and lower GOLD stage compared to smokers [10]. Therefore, in addition to the milder symptoms of never-smoking patients with COPD, they are also in milder stages of the disease according to the GOLD classification [10]. Rani et al. have identified that never-smokers first present to medical care at GOLD stage 2, while most smokers are already in stage 3 at the time of diagnosis [58]. A higher GOLD stage, and implicitly the severity of airway obstruction, is associated with an increased number of emergency presentations and hospitalizations [73].

The annual functional decline, measured by FEV1, is slower in never-smokers (Table A2), suggesting a less accelerated progression of airflow obstruction over time [6].

Functional explorations often show a lower forced vital capacity and a higher prevalence of restrictive patterns in never-smokers. Smokers more frequently show a more pronounced obstruction and a more extensive emphysema [5].

Impulse oscillometry identifies higher resistance and reactance in the airways in never-smokers with COPD exacerbations. Smokers mainly have a lower carbon monoxide diffusion capacity (DLCO), which suggests greater parenchymal destruction [74].

#### 3.5.2. Chest Imaging

Imaging has a central role in characterizing COPD in never-smokers, identifying diverse radiological patterns, and contributing to the diagnosis. Patients with never-smoking COPD have distinct radiological characteristics compared to smokers.

A simple chest X-ray may show signs of pulmonary hyperinflation, which is more common in smokers with COPD than in never-smokers [58].

Emphysematous changes are less common in never-smokers. CT scans in never-smoking COPD patients more commonly show bronchiectasis and post-tuberculous sequelae and less frequently pulmonary emphysema [5,74]. These imaging differences are reflected in symptom patterns: never-smokers tend to have more productive cough and less of the breathlessness that is closely associated with emphysema [5].

Studies using low-dose chest CT studies have shown that never-smokers at increased risk of COPD have reduced pulmonary vessel number, surface area, and volume, indicating that there is early vascular remodeling even in the absence of smoking [75]. It remains to be clarified whether this vascular change even contributes to the pathogenesis of COPD in never-smokers. This vascular remodeling is less obvious than in smokers, probably due to the existence of a different pathophysiological process in COPD in never-smokers [75].

Even in the absence of obvious abnormalities on standard CT, never-smokers with COPD may display subtle bronchial wall thickening detectable through advanced CT analyses. However, these imaging changes are generally less marked than in smokers, and emphysematous destruction is uncommon [76].

#### 3.5.3. Blood and Inflammatory Markers

Never-smokers with COPD often suffer from chronic airway inflammation, likely due to exposures, such as biomass smoke, air pollution, and childhood lung insults, not smoking [15]. Different inflammatory phenotypes are increasingly recognized and correlate with heterogeneity in symptoms, exacerbation risk, and response to inhaled corticosteroids, such as neutrophil-dominant, eosinophil-dominant, or mixed patterns [77]. Never-smokers retain activated macrophages, neutrophils, and lymphocytes in airway and systemic inflammation, driving small-airway fibrosis and airflow limitation, resulting in clinical manifestations such as dyspnea, cough, and sputum [15].

Higher concentrations of neutrophil-related mediators such as IL-8 and IL-17 pathways are associated with poorer lung function, higher cough, and more frequent exacerbations, which indicates that a neutrophilic pattern might contribute to more symptomatic disease independent of smoking status [78]. Conversely, eosinophilic inflammation in COPD correlates with increased exacerbation rates but better response to corticosteroids, and therefore, never–smokers with an eosinophilic pattern might demonstrate frequent flare-ups while benefitting more from anti-inflammatory treatment [79]. In general, these data demonstrate that the type and intensity of airway inflammation, not the smoking exposure directly, are central to symptom burden and clinical course in COPD in never-smokers [77].

In never-smoking patients with COPD, the neutrophil/lymphocyte ratio (NLR) is increased, which serves as a marker of systemic inflammation and disease severity. It correlates negatively with pulmonary function parameters. In never-smokers, NLR is lower than in smokers but remains higher than in healthy individuals. A 2024 study found that never-smokers with COPD had higher eosinophil and lymphocyte counts compared with smokers [80].

Eosinophilic inflammation is important even in never-smokers. A cross-sectional study in 2025 showed that patients who had bronchodilator test reversibility, many of whom were never-smokers, had significantly higher blood eosinophil counts and higher sputum eosinophil levels. Thus, eosinophilic biomarkers help identify asthma-like COPD features and guide therapeutic management in never-smoking COPD patients [81].

The combination of elevated eosinophil counts and FeNO has a predictive value for COPD exacerbations, even in never-smokers. Interestingly, simultaneous increases in FeNO and eosinophils were associated with a protective effect on disease progression [82].

Although FeNO is a well-established biomarker for type 2 airway inflammation in asthma, its application to COPD is still less well-defined and is not adopted as a dominant tool in present-day COPD [83].

Different factors, including corticosteroid treatment, comorbidities, and airway-relevant environmental exposures, can alter FeNO levels and complicate their interpretation in practice [83].

In COPD, FeNO is used primarily as a surrogate marker of eosinophilic/type 2 airway inflammation; however, its association with blood eosinophils is modest and variable, and FeNO alone has not consistently been more powerful in guiding exacerbation risk or inhaled corticosteroid benefit than blood eosinophils [84].

Recent epidemiological and prospective studies have indicated that FeNO could identify patients with eosinophilic inflammation or worse prognosis in COPD, but the specific evidence is inconsistent and insufficiently rich, and therefore its added value, beyond blood eosinophil count for risk stratification and care instruction, is tentative [85]. Accordingly, existing observations and comments on interventions based on guidelines suggest FeNO ideally serves as an adjunctive or exploratory biomarker (not necessarily standard in clinical practice) pending large-scale (if properly studied) studies defining thresholds and clinical utility [86].

In COPD, elevated C-reactive protein, fibrinogen, and leukocyte levels are associated with a higher frequency of future exacerbations, independent of smoking status [87].

The presence of eosinophilic airway inflammation, bronchodilator reversibility, and wheezing in some COPD patients suggests clinical features that overlap with the asthma–COPD overlap (ACO) phenotype [88].

Nonetheless, COPD with eosinophilic inflammation is increasingly regarded as a distinct phenotype within COPD, defined by persistent airflow limitation and a higher prevalence of type 2 inflammatory markers, rather than by a clear history of childhood asthma [89].

By contrast, ACO is usually characterized by asthma diagnosed before 40 years of age, marked bronchodilator reversibility, and frequent coexistence of atopy or elevated IgE levels [90].

In COPD patients without a significant smoking history, the detection of eosinophilia is better viewed as a treatable trait that predicts response to inhaled corticosteroids, rather than as definitive diagnostic evidence of ACO [91].

The GOLD-based literature for 2024–2025 reported blood eosinophil count as the most widely implemented biomarker to inform inhaled corticosteroid (ICS) use in COPD [92].

Elevated blood eosinophil levels are important in patients with COPD and increase the risk of exacerbation and may thus aid in selecting the proper ICS-containing or triple-therapy regimens that may suit them [93].

The increased blood eosinophils characterize an eosinophilic phenotype of COPD, for which ICS (and systemic corticosteroids during exacerbations) are preferred to minimize treatment failure and exacerbation burden [94].

The neutrophil-to-lymphocyte ratio (NLR) is an inexpensive systemic inflammatory marker related to higher inflammation and worse dyspnea and mortality in COPD but not yet included in formal GOLD treatment algorithms [95].

In concert, blood eosinophils and NLR represent potentially therapeutically relevant tools for personalizing COPD management, particularly among phenotypes with elevated type-2 or systemic inflammation, in whom the respective therapeutic response and prognosis differ from standard smoking-related disease [96].

### 3.6. Comorbidities

Comorbidities are more common in never-smoking COPD patients than never-smokers, which influence the course of the disease, its prognosis, and therapeutic management. Fekete et al. analyzed the frequency of comorbidities in never-smokers with COPD versus never-smokers and found that never-smokers had a higher comorbidity burden (3.5 vs. 2.9, *p* < 0.05), likely influenced by genetic factors and environmental interactions [12].

The KOCOSS cohort study found that the prevalence of high blood pressure in COPD was 46.7% in never-smokers, compared with almost 40% in smokers. Large differences were also found in the prevalence of osteoporosis in never-smokers with COPD (10.6%), compared to smokers with COPD (3.5%), which can be explained by the higher percentage of women among never-smokers with COPD [5]. Also, never-smokers with COPD also tend to have a higher body mass index, which leads to metabolic comorbidities [12]. These include diabetes, which increases the risk of hospitalization and mortality, and dyslipidemia, a condition that leads to the onset of a metabolic syndrome [97].

Gastroesophageal reflux is more common in never-smokers with COPD and may contribute to more severe respiratory symptoms and acute exacerbations of COPD [5].

Several respiratory conditions frequently coexist with COPD in never-smokers, including bronchiectasis, asthma, a history of pulmonary tuberculosis, and recurrent respiratory infections in childhood [5]. These associations suggest that a personal history of respiratory disease contributes to the development and progression of airway obstruction [6]. One advantage for never-smoking COPD patients is a lower risk of lung cancer compared with smokers with COPD (0.5% vs. 6.6%), although the risk remains slightly above that of the general population [98].

Studies have also identified an association between COPD in never-smokers and depression, possibly related to the higher proportion of women in this group and the greater burden of comorbidities [5]. Mood disorders, especially those related to stress and anxiety, are common, affecting about one quarter of COPD patients and correlating with poorer clinical outcomes [99].

Sex influences the likelihood of specific comorbidities: asthma and arthritis are more common among women, whereas men show a higher risk of coronary heart disease, gout, and heart failure [100]. Multimorbidity is frequent, and its burden increases with age and with lower socioeconomic status [101].

### 3.7. Treatment

COPD patients usually receive inhaled maintenance therapies similar to those recommended for smokers with COPD, but treatment regimens require individual adaptation depending on the comorbidities and disease-specific characteristics [12]. Inhalation therapy, particularly combinations of long-acting muscarinic antagonists (LAMA) and long-acting beta2-agonists (LABA), has been shown to improve respiratory function in COPD patients, and these benefits are also seen in never-smokers who have undergone lung surgery [102].

Pulmonary rehabilitation has been shown to be highly effective in never-smoking patients with COPD and improves lung function, exercise capacity, and quality of life compared with smokers [62]. The interdisciplinary partnership between family physicians and physiotherapists may increase participation in pulmonary rehabilitation programs, although significant symptom improvements are not always observed in short follow-up periods [103].

An adjuvant therapy for COPD is acupuncture combined with pharmacological treatment, which may improve quality of life and reduce dyspnea [104,105,106,107]. Furthermore, never-smokers with COPD rarely have acute exacerbations, which may influence the treatment durations in such cases [6].

### 3.8. Evolution

Never-smoking patients with COPD have a lower risk of severe exacerbations requiring hospitalization compared to those who have a smoking history. A study led by Nielsen et al., which followed a Danish population cohort for 12 months, found that 12-month mortality was lower in never-smoking COPD patients than in smokers with the disease [10]. Never-smokers who experience COPD exacerbations tend to be older and to have a higher body mass index. In acute exacerbations of COPD in never-smokers, a different inflammatory and clotting profile is suspected, characterized by lower levels of eosinophils and basophils in the blood but higher levels of D-dimer. Moreover, never-smokers with COPD have a lower risk of severe or recurrent exacerbations compared to smokers [98].

Never-smokers with COPD generally have a better quality of life, higher oxygen saturation, a favorable nutritional status, and better lung function compared to smokers [12]. After pulmonary rehabilitation, greater improvements in exercise capacity, dyspnea, and quality of life are observed more frequently in never-smokers [62].

## 4. Conclusions

In conclusion, chronic obstructive pulmonary disease in never-smokers is a distinct clinical entity with different risk factors, characteristics, and disease evolution than in smokers. Although smoking remains the main risk factor for COPD, current data confirm that up to one-third of COPD patients have never smoked.

The pathogenesis of the disease in never-smokers is multifactorial and involves, among other factors, occupational exposures, previous respiratory infections, genetic susceptibility, and socioeconomic determinants. Women, older adults, and individuals living in rural areas or with limited economic resources appear to be the most frequently affected groups.

Clinically, COPD in never-smokers is characterized by a less emphysematous phenotype and milder respiratory symptoms, although patients often present with persistent cough and chronic dyspnea. Pulmonary function generally declines more slowly in these patients than in smokers with COPD.

Paraclinical investigations highlight important pathophysiological differences, supporting a personalized approach to these patients. In a global context marked by decreasing smoking prevalence but persistent environmental and occupational exposures, recognizing COPD in never-smokers becomes essential. A multidisciplinary and individualized approach is key to reducing the burden of this form of COPD and improving patient outcomes.

## Data Availability

Not applicable.

## Abbreviations

The following abbreviations are used in this manuscript:

COPD	Chronic Obstructive Pulmonary Disease
A1ATD	Alpha-1 Antitrypsin Deficiency (alternative notation)
FEV1	Forced Expiratory Volume in 1 Second
FVC	Forced Vital Capacity
FEV1/FVC	Ratio of Forced Expiratory Volume in 1 Second to Forced Vital Capacity
DLCO	Diffusing Capacity for Carbon Monoxide
NLR	Neutrophil-to-Lymphocyte Ratio
FeNO	Fractional Exhaled Nitric Oxide
TNF-α	Tumor Necrosis Factor-alpha
IL-8	Interleukin-8
MMPs	Matrix Metalloproteinases
mMRC	Modified Medical Research Council Dyspnea Scale
CAT	COPD Assessment Test
GOLD	Global Initiative for Chronic Obstructive Lung Disease
KOCOSS	Korea COPD Subgroup Study
VGDF	Vapors, Gases, Dusts, and Fumes
PM2.5	Particulate Matter ≤ 2.5 μm
PM10	Particulate Matter ≤ 10 μm
NOx	Nitrogen Oxides
NO_2_	Nitrogen Dioxide
CO	Carbon Monoxide
HIV	Human Immunodeficiency Virus
TB	Tuberculosis
CD8+ T cells	Cluster of Differentiation 8 Positive T Lymphocytes
OR	Odds Ratio
CI	Confidence Interval
BMI	Body Mass Index
CT	Computed Tomography
QoL	Quality of Life

## Appendix A

**Table A1:** Demographic, Clinical and Radiological Differences Between COPD in Never-Smokers and COPD in Smokers.

Characteristic	COPD—Never-Smokers	COPD—Smokers	References
Sex	More common in women. Biomass fuel exposure more frequent among women	Predominantly in men. Each additional year of age adds to FEV1 decline beyond smoking dose.	[5,12,108,109,110]
Age	Older at the time of diagnosis. Younger in some Asian studies. In severe α1-antitrypsin deficiency, male sex is linked to fixed airflow obstruction.	Younger at diagnosis. Among middle-aged and elderly Chinese adults, being female and a smoker is an independent risk factor for more severe chronic-disease comorbidity patterns.	[6,98,108,111]
Socioeconomic status	Lower educational level. Lower income. More common in rural areas.	Higher educational level. More common in urban areas.	[5,109]
Body mass index (BMI)	Higher BMI. Better QoL and lung function than smokers.	Lower BMI. Obese smokers: worse CAT, more dyspnea/limitations, higher comorbid burden	[5,12,98,108,112,113]
Risk factors	• Biomass fuels; • Outdoor air pollution; • History of pulmonary tuberculosis; • Occupational exposure.	Smoking.	[108,109,114,115,116]
Comorbidities	More frequent: • Hypertension; • Osteoporosis; • Gastroesophageal reflux disease; • Pulmonary tuberculosis; • Bronchiectasis; • Asthma.	More frequent: • Lung cancer; • Cardiovascular diseases.	[5,98,108,115]
Symptom severity	Milder. Lower mMRC and CAT scores.	More severe. Higher mMRC and CAT scores.	[10,12,114]
Chest CT	Less emphysema. More common: • Bronchiectasis; • Post-tuberculosis sequelae.	More frequent, more severe and more extensive emphysema.	[5,6,74,98,108]
Airflow obstruction	Less severe. Higher FEV1/FVC. Better reversibility.	More severe. Lower FEV1/FVC. Reduced reversibility.	[5,12,74,108]
Blood biomarkers	Lower eosinophil and basophil counts. Higher D-dimer levels.	Higher eosinophil and basophil counts.	[98]
Quality of life	Better: • Lower CAT score; • Better oxygen saturation.	Worse: Higher CAT score.	[10,12,59]
Exercise capacity	Similar or slightly better.	Similar or slightly poorer.	[12,108]

**Table A2:** Differences in Disease Evolution and Prognosis Between COPD in Never-Smokers and COPD in Smokers.

Aspect	COPD—Never-Smokers	COPD—Smokers	References
Exacerbation	Lower risk of recurrent and severe exacerbation.	Higher risk of recurrent and severe exacerbation.	[6,11,98]
Pulmonary function	Slower decline in FEV1.	Faster decline in FEV1.	[5,6,12]
All-cause mortality	Lower overall and respiratory mortality.	Higher overall and respiratory mortality.	[6,10,115]
Cardiovascular mortality	Higher than in never-smokers without COPD. Comparable to smokers with COPD.	Highest among all groups.	[115]
Risk of lung cancer	Lower.	Higher.	[98]
Systemic inflammation	Less pronounced.	More prominent.	[6]
Hospitalization	Lower hospitalization rates.	Higher hospitalization rates.	[98]
Treatment response	Better response to inhaled corticosteroids and macrolides.	Reduced response to inhaled corticosteroids and macrolides.	[117]

**Table A3:** Overview of COPD pharmacotherapy differences in smokers versus never-smokers.

Feature	COPD—Smokers	COPD—Never-Smokers	Citations
Overall need for pharmacologic treatment	More severe airflow limitation and symptoms, leading to more intensive pharmacologic management	Generally better lung function and quality of life, so often less intensive regimens	[12]
Use of maintenance inhaled therapy (LABA/LAMA/triple)	More frequent use of dual bronchodilators and triple therapy, reflecting higher symptom burden and exacerbation risk	Lower use of LABA/LAMA and triple therapy; more often managed with simpler bronchodilator regimens	[12]
Changes in medication during follow-up	More frequent medication changes at visits (treatment escalation, regimen switches) than in never-smokers	Fewer medication changes over 12 months	[12]
Response to dual bronchodilation (LABA/LAMA)	Dual therapy (umeclidinium/vilanterol) improves FEV1, dyspnea and rescue use, but benefit is similar in magnitude to former smokers; smoking status has limited impact on bronchodilator response	Never-smokers were not directly studied, but data suggest bronchodilator efficacy is generally preserved when not actively smoking	[118]
Response to triple therapy (ICS/LABA/LAMA)	Triple therapy reduces exacerbations in smokers, but ICS component is relatively less effective due to corticosteroid resistance from smoke exposure	In never-smokers, type-2 inflammation and ICS responsiveness are less impaired, so potential greater relative benefit from ICS when eosinophilic and high-risk	[117,119,120,121]
ICS effectiveness and biology	Cigarette smoke alters glucocorticoid receptor function and airway inflammation, making COPD relatively insensitive to ICS, particularly in current smokers	Never-smokers do not have smoke-induced steroid resistance to the same extent; when eosinophils are elevated, ICS benefit is more predictable	[117,120,121]
ICS risks vs. benefits	Long-term ICS in smokers: pneumonia and fracture risk with more uncertain net benefit in non-eosinophilic disease	Same risks, but risk–benefit balance is more favorable when exacerbation risk and eosinophils are high	[117,121]
